# Research hot spots and trends in endocrine-related adverse events caused by immune checkpoint inhibitors: a bibliometric analysis and visualization research

**DOI:** 10.3389/fendo.2024.1253832

**Published:** 2024-04-15

**Authors:** Jun Zhao, Guangwei Liu, Xue Yang, Chuanzhou Zhang, Bing Han, Man Jiang

**Affiliations:** ^1^ Department of Pharmacy, The Affiliated Hospital of Qingdao University, Qingdao, Shandong, China; ^2^ Department of Gastrointestinal Surgery, The Affiliated Hospital of Qingdao University, Qingdao, Shandong, China

**Keywords:** immune checkpoint inhibitor, endocrine, adverse events, toxicity, bibliometrics, visual analysis

## Abstract

**Background:**

In recent years, with the widespread use of immune checkpoint inhibitors (ICIs) in cancer treatment, the toxicity associated with immunotherapy of ICIs has attracted more attention from scholars. Endocrine toxicity is the most likely immune-related adverse events (irAEs) and is often irreversible, posing a significant clinical treatment challenge.

**Methods:**

In this study, bibliometric methods were used to analyze relevant literature in screening endocrine-related adverse events caused by ICIs in the Web of Science core collection database (WoSCC) and to summarize the status, research hot spots, and future trends in this field.

**Results:**

321 countries, 297 institutions, 365 authors, and 305 journals had published 671 English documents on endocrine adverse reactions of ICIs as of 1 December, 2022. The United States, Japan, and China were the top three countries with the most publications. The University of Texas MD Anderson Cancer Center, Harvard Medical School, and Memorial Sloan Kettering Cancer Center were the top three research institutions in terms of publication output. F Stephen Hodi, from the Dana-Farber Cancer Institute in the United States, contributed the largest number of publications. Frontiers in Oncology, which was the most widely distributed publication in the field. The main keywords or clusters identified that current research hotspots include the management of endocrine-related adverse events, hypophysitis, thyroid dysfunction, type I diabetes mellitus, and the impact of endocrine adverse events on survival of patients in this field.

**Conclusion:**

The basic knowledge structure of the field of endocrine-related adverse events of ICIs, including publication trends, authors, institutions, countries, keywords, journals and publications, and cited documents, was visually analyzed in this bibliometric analysis. The research results comprehensively demonstrated the hot spots and future trends in the research field, as well as its broad prospects, thus providing a reference for the researchers.

## 1 Introduction

Immune checkpoint inhibitors (ICIs) are monoclonal antibodies that achieve immune activation and tumor tissue damage by promoting signaling cascades of T cell function and can improve the immune system’s efficiency in destroying tumor cells (1). The emergence and development of ICIs have brought new concepts and breakthroughs to tumor treatment, as well as unprecedented challenges. ICIs work by non-specific activation of the immune system; consequently, immune-related adverse events (irAEs) of ICIs are an inevitable treatment problem in the use of this class of drugs (2). irAE currently uses more mature cytotoxic T lymphocyte-associated antigen-4 (CTLA-4) inhibitors, programmed cell death receptor-1 (PD-1) inhibitors, and programmed cell death ligand-1 (PD-L1) inhibitors, among others, to varying degrees (3). These irAEs can affect any organ system, range in severity, and can be fatal (4). The endocrine toxicity of this class of drugs is one of the most prevalent irAEs associated with ICIs treatment. The toxicity related to immunotherapy with this class of drugs is a hot topic among scholars (5). The relevant published research (6) includes several summary studies in this field, but bibliometric analysis and visual analysis research have not yet been conducted. Therefore, this article used bibliometric analysis and visual processing to quantitatively and qualitatively evaluate the research trends in the field of endocrine toxicity caused by ICIs, to objectively reveal the field’s research hot spots and development trends, and to provide literature data support for formulating research strategies and directions.

## 2 Method

### 2.1 Data source

The literature on endocrine-related adverse events caused by PD-1/PD-L1 monoclonal antibody drugs was screened in the Web of Science (WOS) core collection database using the following search strategy: keyword((TS=("PD-1"OR"PD-L1 "OR"CTLA-4"OR"Immune checkpoint inhibitors")) AND TS=("adversarial events"OR"side effects"OR"adverse events")) AND TS=("Endocrine"OR"diabetes"OR"thyroid" OR"adrenal gland"OR"hypophysis"). The time was set from the database’s establishment to, 2022-12-01, and 716 articles were retrieved. Papers and review papers were retained, and the language was set to English; eventually, 671 articles were included. The documents were selected as fully recorded with cited references and then exported in.txt format.

### 2.2 Data processing

The research used CiteSpace 6.1.R6 with the following settings. Time slicing: January, 2004 to 1 December, 2022 (the first related research was published in, 2004), with one-year time slices. Node types: author, institution, keywords. If the node was the author: threshold (top N per slice) = 25, pruning = none; if the node was the institution: threshold (top N per slice) = 25, pruning = none; if the node was the keyword: threshold (top N per slice) = 25, pruning = pathfinder + pruning the merged network. Visual analysis was performed based on the parameter settings of each node to generate a knowledge map of authors, institutions, countries, and collaborations in the research field of endocrine-related adverse events caused by ICIs; co-occurrence, emergence, and clustering of keywords; a knowledge graph; and a timeline chart for keywords.

**Figure 1:**
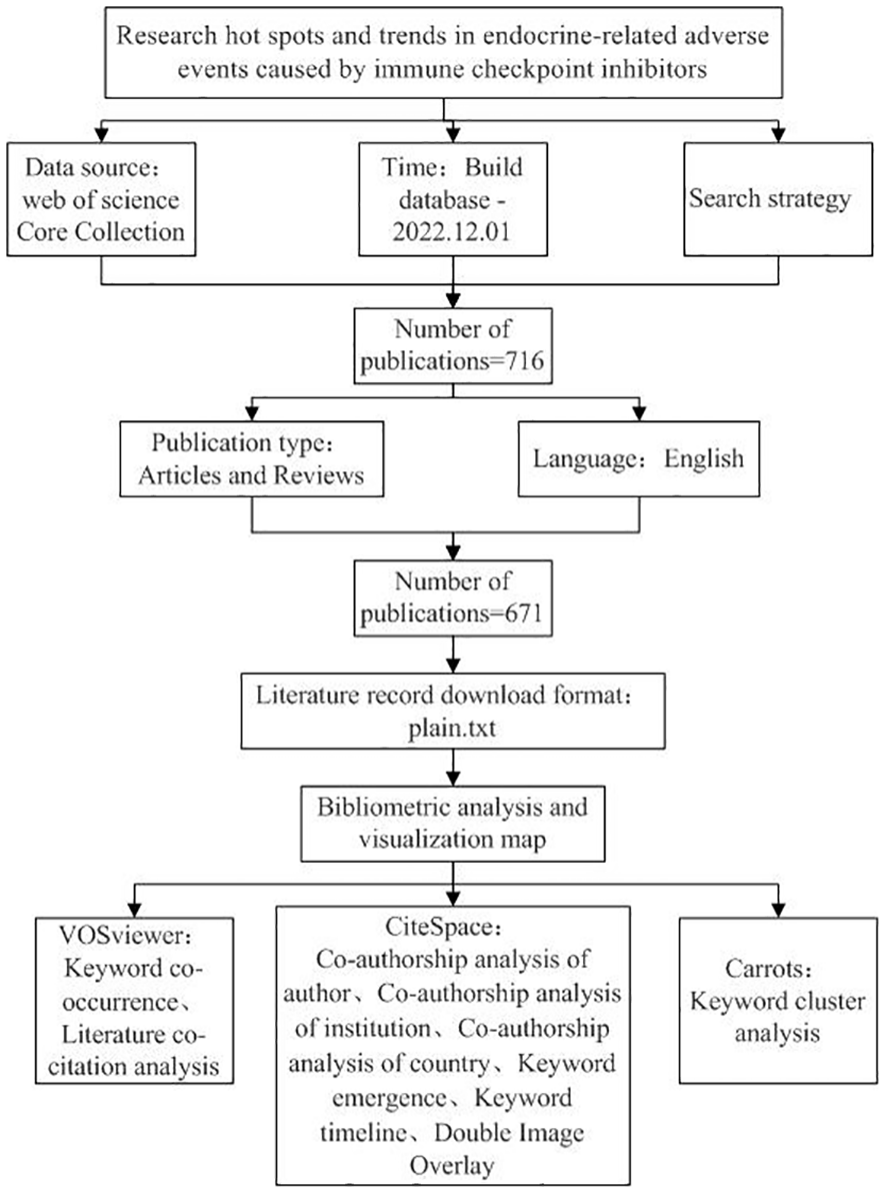
Frame flow diagram shows the detailed selection criteria and bibliometric analysis steps in this study.

The documents retrieved from the WOS database were exported in plain text format with complete records and cited references as the contents. The data were imported into the VOSviewer 1.6.18 software, the calculation method was set to full calculation, and thresholds were established following the various analysis projects. After creating a visual map, the cooperation network was analyzed. **Figure 1** depicts the research process and its essential steps.

## 3 Results

### 3.1 Posting trends

**Figure 2:**
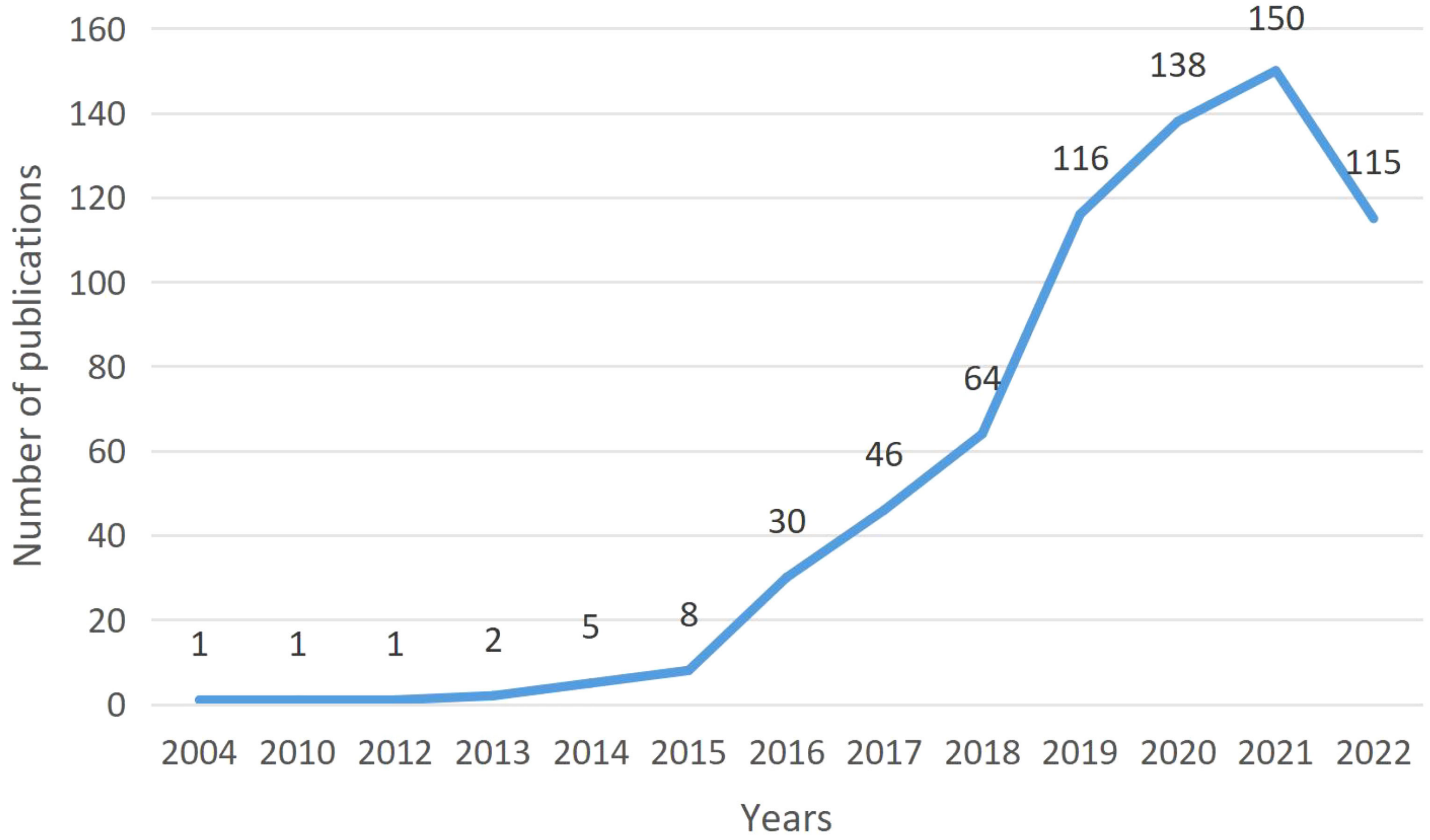
The number of publications per year from, 2004 to, 2022.

The first study on ICIs associated endocrine irAEs was published in, 2004. F. Monzani, an Italian scientist, published an article on thyroid autoimmunity and dysfunction associated with type I interferon therapy in “Clinical and Experimental Medicine.” According to the publication trend (**Figure 2**), the annual publication volume had fluctuated, and then, in, 2018, it began to accelerate. After, 2019, the annual publication volume exceeded 100, reaching 143 in, 2021. In, 2022, no all data was included, and 115 articles were published. The trend was expected to be slightly lower than in, 2021.

### 3.2 Author collaboration network

**Figure 3:**
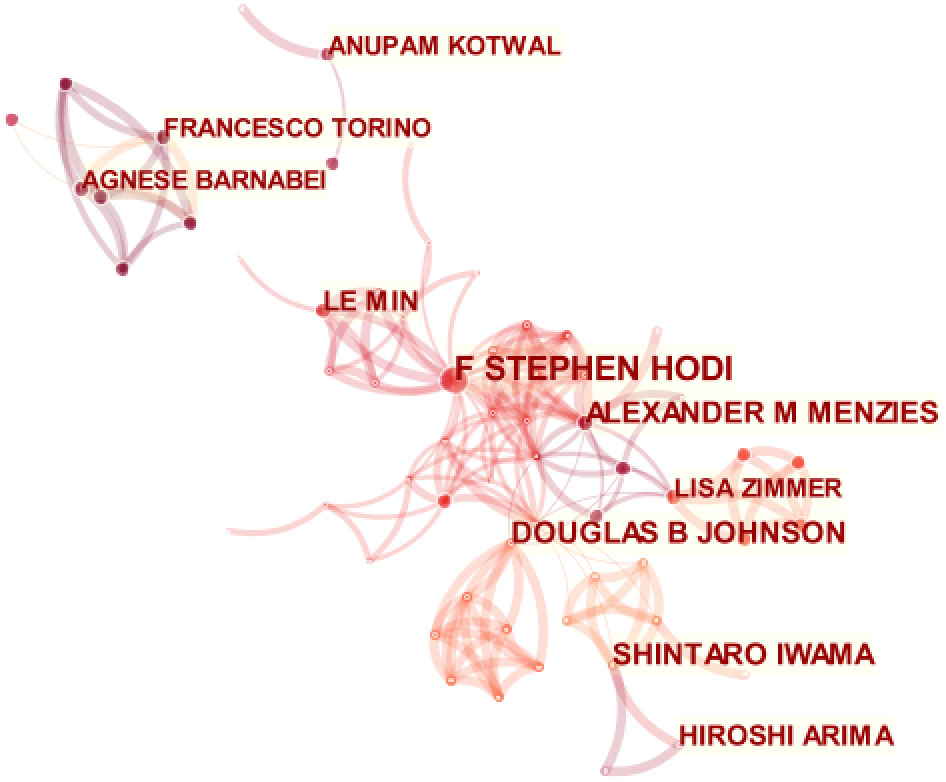
Map of author cooperation network in this field, connecting lines stand for author interaction.

Citespace was used to analyze an article’s author, leading to **Figure 3**. The figure has 365 nodes and 801 connections, with a network density of 0.0121. Each node in the figure represents an individual author. The greater the node radius, the greater the number of articles published. The number of articles between each pair of nodes, represented by a connecting line, signifies the connection or collaboration between the authors. The cooperative relationship is closer when the connecting line is thicker. According to the findings, the authors in the field have established six collaborative networks. The research team led by F. Stephen Hodi is the largest. The team mainly studies the therapeutic effect and safety of nivolumab and ipilimumab in patients with advanced melanoma, as well as ipilimumab treatment and the effects and safety of metastatic melanoma. The second-largest team is led by Douglas B. Johnson, whose research focuses on the multi-system toxicity caused by ICIs, including endocrine toxicity, neurotoxicity, and cardiotoxicity, as well as the long-term effects of these toxicities on the treated population. Shintaro Iwama heads the third-largest team. This team mainly focuses on the clinical characteristics, treatment, and biomarker identification of endocrine dysfunction induced by ICIs, particularly in thyroid and pituitary dysfunction. Their research is relatively more comprehensive. **Table 1** displays the top ten authors in the research field.

**Table 1:** The top ten authors in the research fields from, 2004-2022.

Rank	Author	Count	Year
1	F Stephen Hodi	10	2017
2	Douglas B Johnson	7	2015
3	Shintaro Iwama	7	2014
4	Alexander M Menzies	7	2017
5	Le Min	6	2018
6	Hiroshi Arima	6	2018
7	Agnese Barnabei	5	2013
8	Lisa Zimmer	5	2016
9	Anupam Kotwal	5	2020
10	Francesco Torino	5	2013

### 3.3 Institutional cooperation network

**Figure 4:**
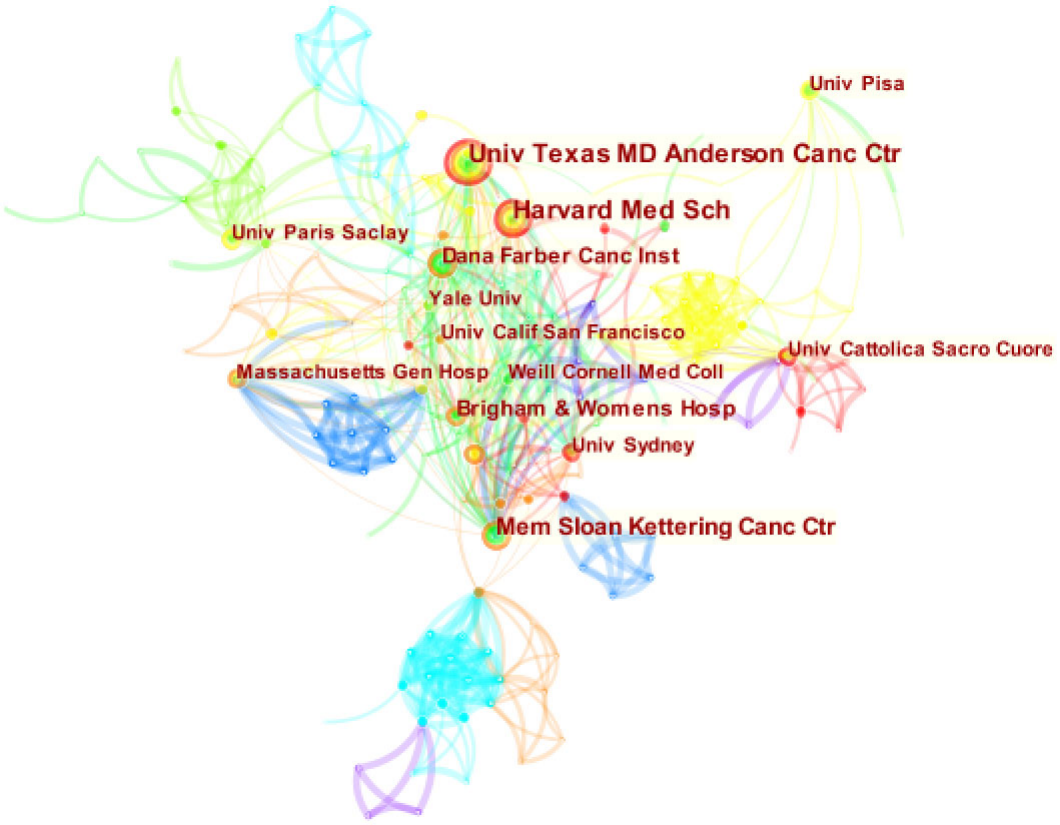
Map of institutional cooperation network in this field. Circle size means the number of institutions published articles; the connecting lines stand for mutual communication and interaction.

**Figure 4** is obtained by analyzing the publishing institutions in the study’s included literature using Citespace. The figure contains 297 nodes and 805 connections, with a network density of 0.0183. The study included 297 institutions, nine of which published more than ten articles (**Table 2**). The University of Texas MD Anderson Cancer Center was the institution with the most publications (25), followed by Harvard Medical School (22) and Memorial Sloan-Kettering Cancer Center (19). The issuing institutions comprise cancer research centers, medical schools, and comprehensive universities. Moreover, there are cooperative ties between institutions. It may be because most American research institutions make cooperation more convenient. Centrality is a metric used to evaluate the significance of a network node’s position. A value ≥ 0.1 indicates that it plays a significant role in the field’s evolution, reflecting the current hot research direction (7). The centralities of the three research institutions, Memorial Sloan-Kettering Cancer Center, University of Sydney, and Weill Cornell Medical College are all greater than 0.1, indicating that their research is vital.

**Table 2:** The top ten institutions in the research fields from, 2004-2022.

Rank	Institution	Country	Count	Centrality	Year
1	University of Texas MD Anderson Cancer Center	USA	25	0.08	2017
2	Harvard Medical School	USA	22	0.02	2019
3	Memorial Sloan-Kettering Cancer Center	USA	19	0.19	2014
4	Dana-Farber Cancer Institute	USA	16	0.07	2017
5	Brigham and Women’s Hospital	USA	14	0.01	2017
6	University of Pisa	Italy	11	0.03	2004
7	The University of Sydney	Australia	11	0.14	2017
8	University of California, San Francisco	USA	10	0.08	2017
9	Massachusetts General Hospital	USA	10	0.02	2019
10	Weill Cornell Medical College	USA	9	0.19	2014

### 3.4 Analysis of national or regional cooperation networks

**Figure 5:**
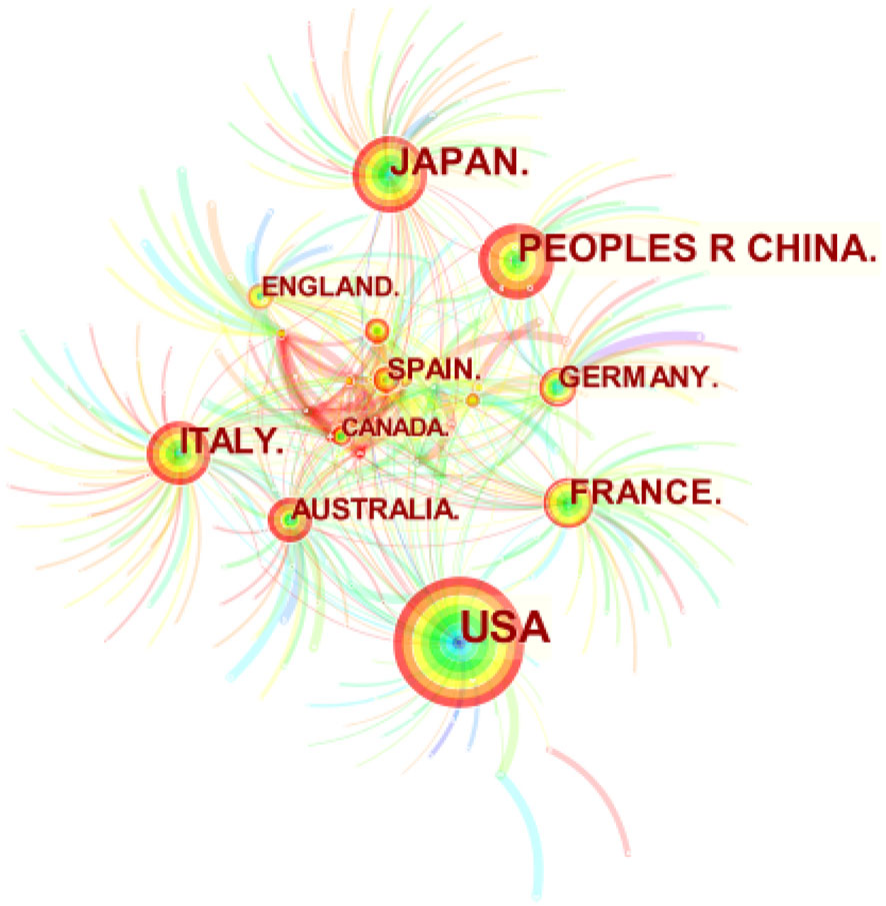
Map of national or regional cooperation network in this field. Circle size means the number of published articles.

As shown in **Figure 5**, Citespace was used to determine the number of publications by country or region in the study’s documents. The figure shows that 321 countries have published relevant documents in the field. The figure depicts 608 connections, with a network density of 0.0118. The United States has published the most research in the field (223), followed by Japan (80), China (75), Italy (64), and France (47). **Table 3** lists the ten countries or regions with the most published articles. The United States, Japan, and China play a significant leadership role in the field. The centrality indicates that research significance in the United States and Japan is relatively high, indicating that researchers from these countries have published many influential publications. Meanwhile, China began research in the field relatively late. However, its research activity has increased in recent years, as its research quality still has room for improvement compared to the top two countries. -

**Table 3:** The top ten countries or regional in the research fields from, 2004-2022.

Rank	Country	Count	Centrality	Year
1	USA	223	0.43	2010
2	Japan	80	0.43	2014
3	Peoples R China	75	0.13	2017
4	Italy	64	0.45	2013
5	France	47	0.33	2016
6	Australia	32	0.18	2015
7	Germany	31	0.19	2013
8	Spain	28	0.01	2016
9	England	27	0.12	2015
10	Canada	18	0.08	2017

### 3.5 Keywords

#### 3.5.1 Keyword co-occurrence

**Figure 6:**
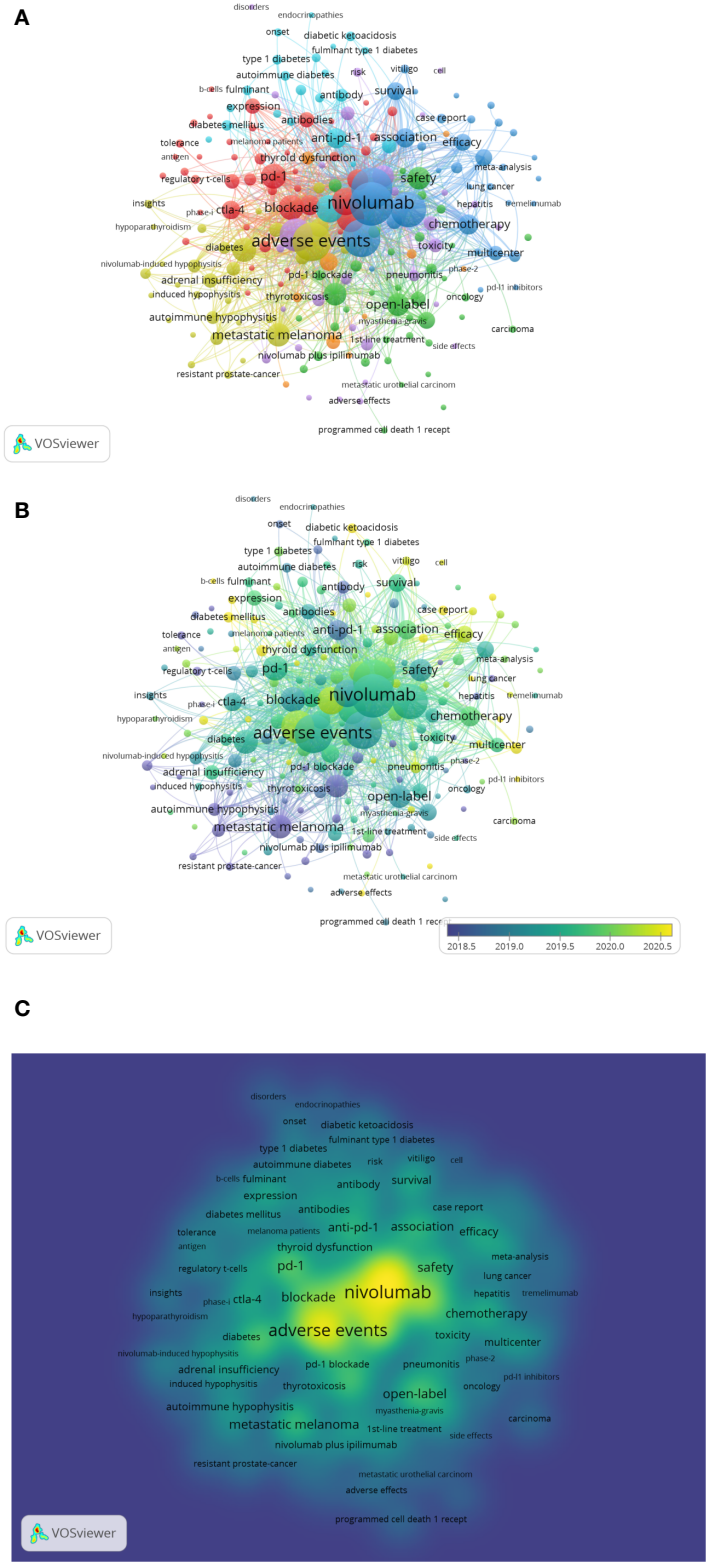
**(A)** Keyword co-occurrence analysis of network knowledge graph. The keywords fell into seven clusters based on colors: Cluster 1, 2, 3, 4, 5,6 and 7 are, respectively red, green, blue, yellow, purple, light blue and orange. The node size denotes the occurrence frequency. **(B)** Distribution of keywords according to the mean frequency of appearance, the different colours indicate the relevant year of publication. Yellow keywords came later than green keywords. **(C)** Keyword co-occurrence density map based on VOSviewer, indicates the occurrence frequency of keyword.

Keywords are a high-level summary of the paper’s topic. Co-occurrence analysis uses keywords as nodes and tailors them to form a map of nodes and connections. Vosviewer was used to generate keyword co-occurrence network diagrams and overlay visualization diagrams for visual analysis. With a minimum of five occurrences as the screening criterion, **Figure 6A**’s co-occurrence network reveals that of the 1,753 keywords, 211 were screened out and divided into seven clusters. Cluster 1 (44 items, red) is an immune checkpoint inhibitor related to research on the pathogenesis and biomarkers of endocrine toxicity. Cluster 2 (38 items, green) is a clinical trial related to ICIs. Cluster 3 (34 items, blue) describes the clinical characteristics of immune checkpoint inhibitor-induced endocrine toxicity, including research on treatment and prognosis. Cluster 4 (32 items, yellow) is related to adrenal and pituitary dysfunction caused by ICIs. Cluster 5 (31 items, purple) is related to renal toxicity, liver toxicity, and pulmonary toxicity caused by ICIs, including related studies. Cluster 6 (22 items, light blue) studies diabetes-related adverse events caused by anti-PD-1 and PD-L1 monoclonal antibodies. Cluster 7 (10 items, orange) is a study on anti-PD-1 monoclonal antibody-induced thyroid dysfunction, including related research. The keyword overlay visualization diagram (**Figure 6B**) incorporates time factors. Various colors correspond to the year in which the keyword first appeared. The earlier the keyword appears, the greener the color; the later the keyword appears, the redder the redder it is. **Figure 6C** depicts the keyword co-occurrence map densities. Each point in the density visualization is colored according to the item’s density at that point. The greater the density, the closer the color is to red, while the lower the density, the closer to blue. This map can determine the knowledge and research density in the field.

#### 3.5.2 Keyword cluster analysis

**Figure 7:**
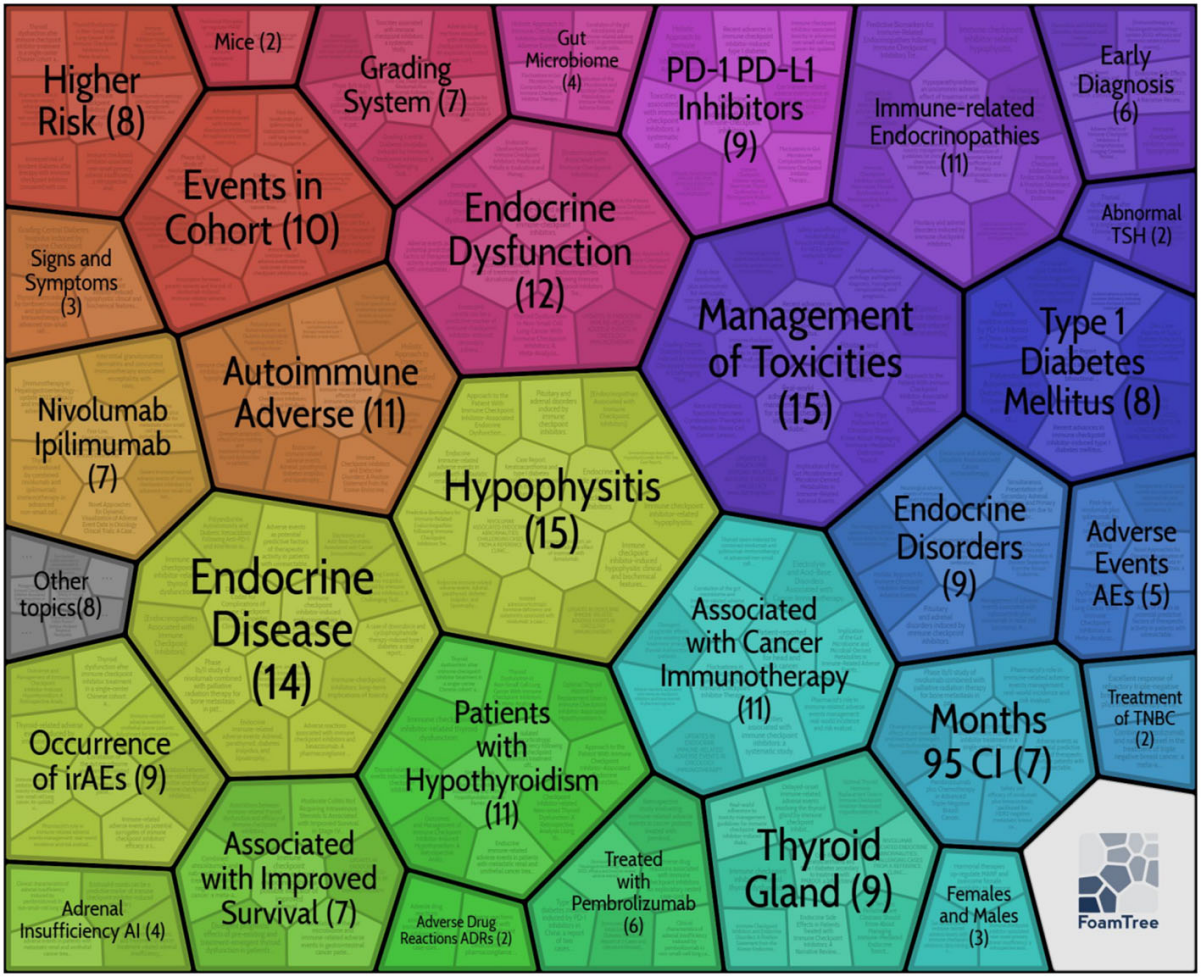
Keywords Cluster analysis atlas based on the carrot system, the numbers represent the frequency of keyword clustering.

**Figure 7** depicts a keyword clustering diagram created with Carrot2. As shown in the figure, current research hotspots include the management of endocrine-related adverse events, hypophysitis, thyroid dysfunction, type I diabetes mellitus, and the impact of endocrine adverse events on survival of patients are also a research focus in this field.

#### 3.5.3 Keyword emergence

**Figure 8:**
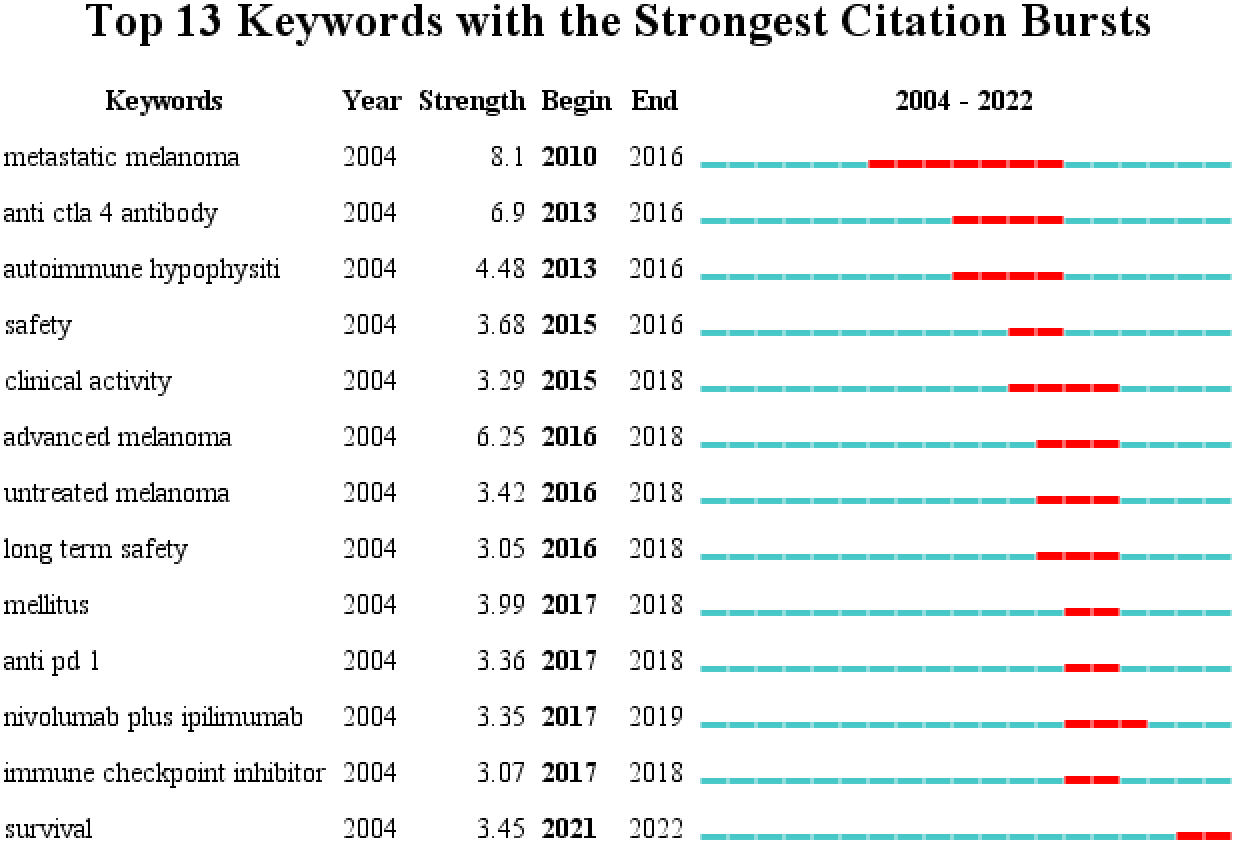
The top 13 keywords with the most bursts. The year represented by the red line indicates the time period when the keyword was mainly influential.

Keyword emergence refers to a significant increase in keyword frequency in a short period. Understanding the research that has received significant attention during this period can be used to determine the research hot spots and frontiers in the field (8). **Figure 8** depicts the emergence analysis of keywords in research literature regarding endocrine-related adverse events caused by ICIs. Setting the parameters γ[0,1] = 1.0 and minimum duration = 1 and obtained 13 emergent words. The results show that research began in, 2004, and additional related research did not emerge until, 2010. Disease-related research focuses primarily on melanoma, drug-related research on anti-CTLA-4 monoclonal antibodies and anti-PD-1 monoclonal antibodies, and adverse events research on autoimmune hypophysitis and diabetes. In the past five years, research has primarily focused on diabetes-related adverse events caused by ICIs, endocrine-related adverse events caused by combined nivolumab and ipilimumab, and the impact of such adverse events on patient survival.

#### 3.5.4 Keyword timeline chart

**Figure 9:**
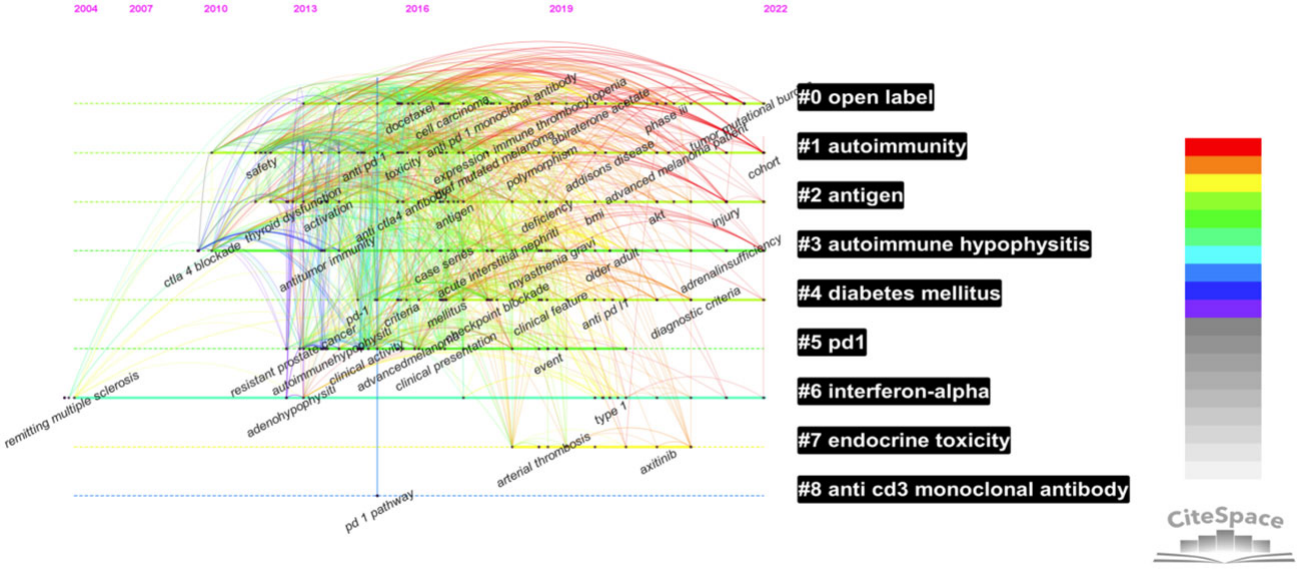
Keyword timeline visualization chart based on Citespace.

By selecting “timeline”, a timeline map of document clustering was drawn, and the period and correlation of clustering were visually analyzed, as shown in **Figure 9**. Clusters #5, #7, and #8 have ceased to evolve. In contrast, clusters #0 (open-label), #1 (autoimmunity), #2 (antigen), #3 (the research fields represented by autoimmune hypophysitis), #4 (diabetes mellitus), and #6 (interferon-alpha) have a relatively long period and continue to the present day; hence, they are enduring research hot spots.

### 3.6 Top ten cited documents and co-cited documents

#### 3.6.1 Top ten cited documents

The top 10 cited documents are listed (**Table 4**). The most cited document (1241 times) is the article “Immune-related adverse events with Immune checkpoint blockade: a comprehensive review” (9) by Michot, J.M., published in “European Journal of Cancer” (IF =10.002) in, 2016. The article systematically reviewed the mechanisms of irAEs of specific immune checkpoint molecules cytotoxic T lymphocyte-associated antigen-4(CTLA-4), programmed cell death protein (PD-1)and its ligand PD-L1, and the putative relationships between immunodystoxicity and antitumor efficacy. The relevant content of this article has become the basis for management guidelines.

**Table 4:** Statistical table of the top ten cited literatures in the field.

Rank	Title	First author	Publication Year	Total Citations	Average per Year
1	Immune-related adverse events with immune checkpoint blockade: a comprehensive review	Michot, J. M	2016	1241	177.29
2	Managing toxicities associated with immune checkpoint inhibitors: consensus recommendations from the Society for Immunotherapy of Cancer (SITC) Toxicity Management Working Group	Puzanov, I	2017	997	166.17
3	Safety Profile of Nivolumab Monotherapy: A Pooled Analysis of Patients With Advanced Melanoma	Almazor, M. E	2017	692	115.33
4	Association of tumour mutational burden with outcomes in patients with advanced solid tumours treated with pembrolizumab: prospective biomarker analysis of the multicohort, open-label, phase 2 KEYNOTE-158 study	Marabelle, Aurelien	2020	657	219
5	Safety profiles of anti-CTLA-4 and anti-PD-1 antibodies alone and in combination	Kao, Steven	2016	606	86.57
6	Management of toxicities of immune checkpoint inhibitors	Spain, Lavinia	2016	525	75
7	Incidence of Endocrine Dysfunction Following the Use of Different Immune Checkpoint Inhibitor Regimens A Systematic Review and Meta-analysis	Barroso-Sousa	2018	471	94.2
8	Myocarditis in Patients Treated With Immune Checkpoint Inhibitors	Mahmood, Syed S	2018	425	85
9	Pituitary Expression of CTLA-4 Mediates Hypophysitis Secondary to Administration of CTLA-4 Blocking Antibody	Awadalla, Magid	2014	399	44.33
10	Cutaneous, gastrointestinal, hepatic, endocrine, and renal side-effects of anti-PD-1 therapy	Hofmann, Lars	2016	393	56.14

#### 3.6.2 Literature co-citation network

**Figure 10:**
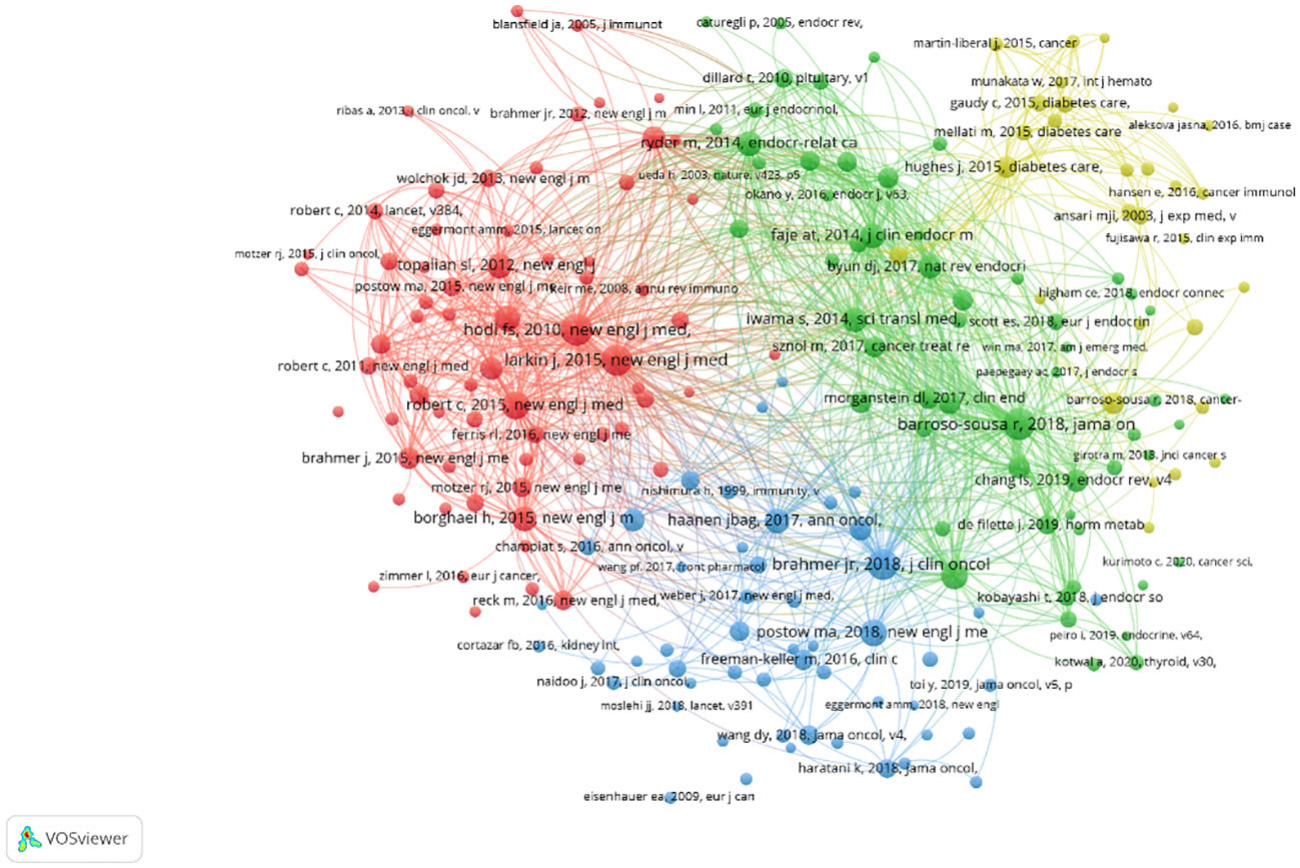
Document co-citation network diagram. The different colours of the nodes represent different types of documents, with larger nodes meaning articles that are collectively cited more frequently.

Vosviewer was used to analyze co-cited documents. A total of 12,738 co-cited documents were extracted. At least 20-times cited documents were used for data extraction, and 205 of these documents were obtained. A co-cited document network was generated (**Figure 10**). The documents were divided into four clusters according to the color blocks. **Table 5** displays the ten documents with the most citations. Most cited documents were published in high-level journals; six of them were published in the magazine “New English J Med”, with an impact factor of 176.079. All cited documents were likewise separated into four clusters. Cluster 1 (67 items, red) included comprehensive high-level medical journal research; Cluster 2 (58 items, green) included research on journals related to endocrinology; Cluster 3 (51 items, blue) included research on journals related to oncology; and Cluster 4 (29 items, yellow) included research on journals related to diabetes.

**Table 5:** Statistical table of top ten co-cited literature in this field.

Rank	Title	First Author	Year	Journal	IF(2022)	Citations
1	Improved survival with ipilimumab in patients with metastatic melanoma	Hodi FS	2010	New Engl J Med	176.079	175
2	Incidence of Endocrine Dysfunction Following the Use of Different Immune Checkpoint Inhibitor Regimens: A Systematic Review and Meta-analysis	Barroso-sousa R	2018	Jama Oncology	33.006	167
3	Management of Immune-Related Adverse Events in Patients Treated With Immune Checkpoint Inhibitor Therapy: American Society of Clinical Oncology Clinical Practice Guideline	Brahmer Jr	2018	J Clin Oncol	50.717	161
4	Combined Nivolumab and Ipilimumab or Monotherapy in Untreated Melanoma	Larkin J	2015	New Engl J Med	176.079	157
5	Pembrolizumab versus Ipilimumab in Advanced Melanoma	Robert C	2015	New Engl J Med	176.079	128
6	Immune-Related Adverse Events Associated with Immune Checkpoint Blockade	Postow Ma	2018	New Engl J Med	176.079	125
7	Antibody-mediated thyroid dysfunction during T-cell checkpoint blockade in patients with non-small-cell lung cancer	Osorio JC	2017	Ann Oncol	51.769	124
8	Nivolumab in previously untreated melanoma without BRAF mutation	Robert C	2015	New Engl J Med	176.079	111
9	Nivolumab versus Docetaxel in Advanced Nonsquamous Non-Small-Cell Lung Cancer	Borghaei H	2015	New Engl J Med	176.079	105
10	Ipilimumab-induced hypophysitis: a detailed longitudinal analysis in a large cohort of patients with metastatic melanoma	Faje At	2014	J Clin Endocr Metab	6.134	104

### 3.7 Leading journals

A total of 305 journals have published research-related articles. **Table 6** displays the ten journals with the highest number of research publications. One hundred forty-six articles have been published, accounting for 21.40% of all literature. Most of JCR journals are Q1 or Q2. The journal with the most published articles is “Frontiers in Oncology” (20 articles, 2.93%), focusing primarily on cutting-edge tumor treatment research.

**Table 6:** The top 10 leading journals with the most published papers in the field.

Journal of Publication	Count	Percentage	IF	JCR(2022)
Frontiers in Oncology	20	2.93%	5.738	Q2
Cancers	18	2.64%	6.575	Q1
Oncologist	17	2.49%	5.837	Q2
Journal of Clinical Endocrinology & Metabolism	15	2.20%	6.134	Q1
Cancer Immunology, Immunotherapy	14	2.05%	6.63	Q1
European Journal of Cancer	14	2.05%	10.002	Q1
Journal for ImmunoTherapy of Cancer	14	2.05%	12.469	Q1
Journal of Oncology Pharmacy Practice	14	2.05%	1.416	Q4
Frontiers in Immunology	10	1.47%	8.786	Q1
Thyroid	10	1.47%	6.506	Q1

### 3.8 Double figure overlay

**Figure 11:**
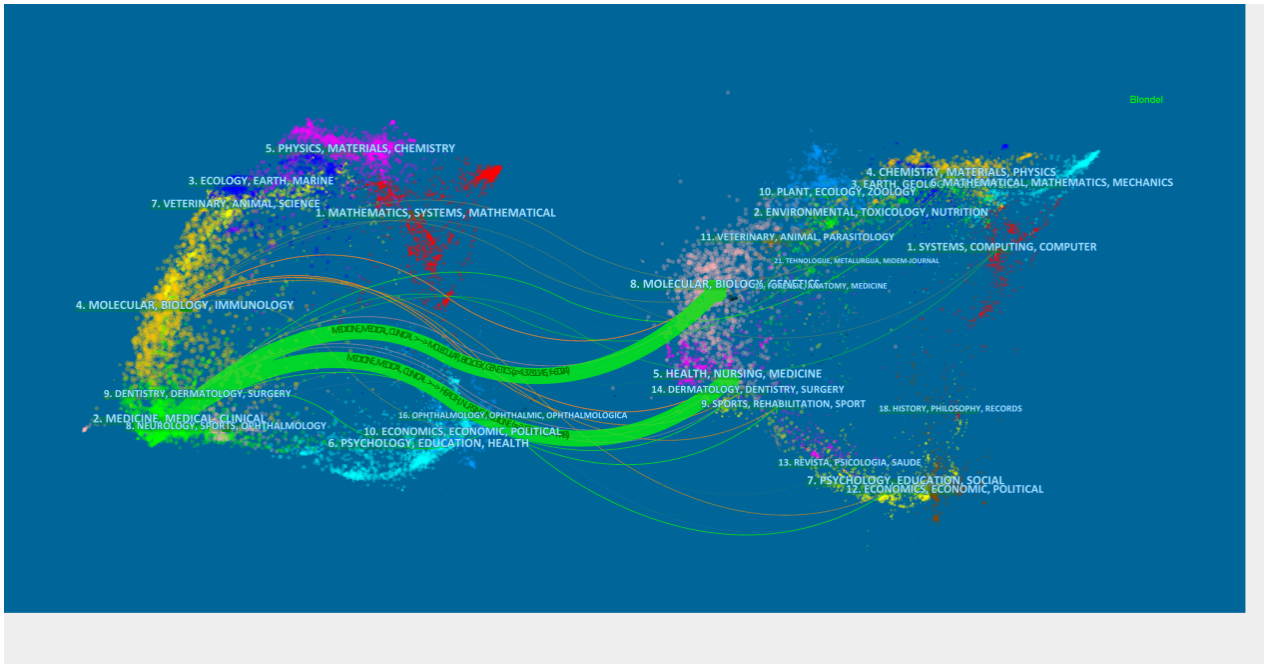
Double graph overlay of relevant literature on endocrine-related adverse events caused by ICIs. On the left were the citing journals, on the right were the cited journals, and the colored path represented the citation relationship.

A double-figure overlay aims to reveal overall scientific contributions and interactions between journals (10). Publications and citations in the field can be described at the subject level. A citation bimap was constructed using Citespace’s bimap overlay function. The left half represents the citing document, the right half denotes the cited document, and the curve represents the citation correlation line. The citing document is primarily affected by the cited document. This connection illustrates the flow and interconnectedness of knowledge in different research fields (**Figure 11**). By Z-score, medicine, medical, and clinical are the most frequently covered record fields, and research is affected by these fields.

## 4 Discussion

### 4.1 Research status and main results

ICIs are monoclonal antibodies that bind and inhibit CTLA-4 or PD-1 and its ligand PD-L1 to target two key signaling pathways related to T cell activation and exhaustion. ICls, such as nivolumab, pembrolizumab, and ipilimumab, have been approved for treating numerous types of cancer in various combination regimens and are currently the cornerstone of cancer treatment. ICl-induced toxicity is autoimmune and known as irAEs, which may unpredictably affect any organ system (11). irAEs may manifest as endocrine disorders, including thyroid, pituitary, adrenal, and pancreatic disorders (12). In this study, we used data mining and visualization technology to draw a knowledge map of research on endocrine-related adverse events caused by ICIs. We comprehensively searched for literature on this topic published in the WOS core collection database before 1 December, 2022. We included a total of 671 publications for bibliometric analysis.

The number of publications in different years reflects, to some extent, the degree of researchers’ attention to the field. In, 2004, Italian scientist F. Monzani published the first report on thyroid autoimmunity and dysfunction associated with type I interferon therapy in Clinical and Experimental Medicine. The study revealed that the side effects of type I interferons lead to multiple changes in thyroid function, some of which are related to autoimmunity, with hypothyroidism occurring more frequently than hyperthyroidism. It has been confirmed that CTLA-4 gene polymorphisms are associated with thyroid dysfunction. From that point forward until, 2014, only 10 papers were published in eleven years. No relevant research results were published for many consecutive years, especially from, 2005 to, 2009. Since the first PD-1 inhibitor pembrolizumab was approved for market by the US Food and Drug Administration (FDA) in, 2014, Many types of ICIs have been approved and applied to the treatment of various tumors worldwide. An annual rise in the frequency of drug-related adverse reactions has coincided with the addition of new indications for ICIs in cancer therapy and the growing patient population. The negative consequences of irAEs on patients are now receiving more attention (13). Consequently, since, 2015, there has been a significant increase in the field of irAE research. The, 2018 Nobel Prize in Medicine or Physiology was awarded jointly to James P. Allison and Tasuku Honjo “for their discovery of cancer therapy by inhibition of negative immune regulation” (14). Since then, it has further boosted the research heat in this field, leading to a more significant increase in the number of studies since, 2018. As of, 2021, the annual publication volume has reached 150 articles. Our research has found that as of December 1, 2022, 115 articles have been published in this field. Although the number of publications has slightly decreased compared to last year, it is still at a relatively high level. We believe that with the increasing clinical application of ICIs, research in this field will continue to receive widespread attention and remain a hot topic for future research.

### 4.2 Research hot spots and cutting-edge trends

Keyword clustering illustrates the direction of research hotspots in this field, whereas the timeline view depicts the evolution of related hotspots over time. Combining keyword co-occurrence with highly cited papers makes it possible to identify and detect research hotspots and frontiers with greater precision. In, 2004, research in this field began. Since, 2010, more research on metastatic melanoma has been published. Anti-CTLA-4 monoclonal antibodies and anti-PD-1 monoclonal antibodies have been the subject of many safety studies in this field. Nivolumab, ipilimumab, and pembrolizumab have become popular research topics in this field. After many safety studies on the use of single drugs emerged and grew increasingly comprehensive, studies on the safety of the combination or sequential use of anti-PD-1 and anti-CTLA-4 drugs appeared immediately (15, 16). In a phase 3 study, researchers compared the effectiveness of the combination of ipilimumab and nivolumab to that of either antibody alone. This combination elicited a more robust response. However, immune-related toxicities occur more frequently and at a higher grade than with either agent alone (17). In a separate exploratory study, the combination of ipilimumab and nivolumab was more effective than nivolumab alone regarding progression-free survival and overall survival landmarks (18). In, 2015, regulatory approval was granted for combination immunotherapy (19). However, due to the higher risk of toxicity associated with combination therapy, researchers are very interested in identifying the subgroups of patients most likely to benefit from combination therapy. So, they weigh the therapeutic efficacy of combination therapy with ICIs against the incidence of irAEs. Since, 2021, the patient population that may benefit from combination therapy has become a focal point of research in this field.

Comprehensive studies of adverse effects at different endocrine sites have started to appear and have reached their current degree of maturity as a result of the continual development of safety studies. According to reports, thyroid and pituitary toxicity are the most common endocrine toxicities of this class of drugs (20) and are also the most studied by researchers. Diabetes and adrenocortical insufficiency are uncommon endocrine toxicities associated with ICI treatment (21), but diabetic ketoacidosis (DKA) and adrenal crisis are frequently life-threatening. This study found that most of the field’s research focuses on autoimmune hypophysitis resulting from ICIs, diabetes resulting from ICIs, and thyroid dysfunction resulting from ICIs.

The treatments most commonly associated with immune-related hypophysitis are anti-CTLA-4 monotherapy, anti-CTLA-4 and anti-PD-1 combination therapy, and anti-PD-1 or anti-PD-L1 monotherapy (22). Hypophysitis secondary to tumor immunotherapy is an emerging area of clinical research. It may be attributable to its non-specific symptoms and signs, which are frequently overlooked and can have severe consequences. Currently, cases of delayed hypophysitis occurring several months after the cessation of ICIs have garnered much scholarly interest (23, 24).

This study found that the study of diabetes-related adverse events caused by ICIs has become a hot topic. Diabetes caused by ICIs has been reported to be more prevalent following treatment with PD-1/PD-L1 inhibitors (25, 26). The disease progresses rapidly to islet failure, and the damage to islet B cell function is frequently irreversible (27). Most cases of this type of diabetes require daily insulin treatment over the long term (28). If diabetes caused by ICIs is not diagnosed and identified promptly, the risk of diabetic ketoacidosis (DKA) is elevated (21). In most ICIs-related diabetes cases (70%) described to date, DKA is the underlying cause. Consequently, this type of adverse reaction has also become a popular topic of study among academics.

There is a reported 6%–20% incidence of thyroid-related injuries with ICIs, including hyperthyroidism, hypothyroidism, thyroiditis, thyrotoxicosis, and thyroid storm, especially with the use of anti-PD-1 or anti-PD-L antibodies (29). It has been reported that PD-1/PD-L1 inhibitors cause a more significant proportion of hyperthyroid patients than CTLA-4 inhibitors. The combined use of ICIs will increase the proportion of hyperthyroid patients by at least threefold (30, 31). Consider the positive aspects of thyrotoxicity, thyroid irAEs are associated with better cancer outcomes, improved survival, and a better prognosis for patients who experience them (32, 33). It may be due to the effective activation of the immune system (34), and the specific mechanism has become the focus of research by scholars. Historically, most endocrine adverse reactions were caused by the involvement of a single gland. However, with the gradual increase in clinical use of ICIs, reports of involvement of two or more glands are no longer uncommon. According to studies, the combination of thyroid and pancreatic injuries is the most prevalent multi-gland injury (35). Patients receiving ICIs must continue to monitor these endocrine-related events.

ICI-related endocrine adverse reactions are severe but quite manageable. Although they rarely lead to treatment discontinuation, many irAEs are irreversible (36). If endocrine irAEs can be effectively managed, the patient’s prognosis will be drastically improved, and the patient’s survival time will be extended. However, irreversible irAEs continue to have an impact on the quality of life of patients. This study found that the management of endocrine irAEs and the study of the survival of patients with irreversible endocrine adverse events may be future research hotspots in the field. Clinicians should focus on monitoring endocrine adverse reactions, the balance between endocrine adverse reactions and patient prognosis, and the accurate assessment of the balance between patient prognosis and the harm of adverse reactions to those who will benefit. In addition to strong interdisciplinary collaboration, as the number of patients receiving ICIs rises, it is critical that researchers in this field aggressively pursue efficient ways to predict biomarkers of the risk of endocrine adverse effects (37).

### 4.3 Limitations

Certainly, this paper has some limitations. The data was only retrieved from the WOSCC database, and the research literature of some countries may have been omitted. Furthermore, our study only included relevant literature in English, and studies published in other languages were excluded, which could cause a certain degree of bias in the analysis. Nonetheless, WOS remains the most commonly used database for scientometric analysis, and English is today’s international lingua franca. In addition, recently published high-quality studies may not have received the attention they deserved due to citation delays and need to be updated in subsequent studies. Ultimately, we believe that these limitations may slightly impact the results, but will not have a significantly impact on the main trend in the field.

## 5 Conclusion

This study analyzed trends and hot spots in ICIs associated endocrine irAEs research. Based on the results, we believe that future research hotspots will mainly focus on the following aspects: Firstly, it is critical to accurately assess the balance between efficacy and adverse effects in patients with ICIs to facilitate the identification of these endocrine irAEs events and prognostic or survival analysis studies. Secondly, the continuous deepening and expansion of research on new irAEs, with the application of new ICIs drugs and combination therapy. Thirdly, conducting cohort studies to understand risk factors and patterns of onset. These future hotspots are crucial to promoting greater advancing on irAEs, which is where the potential of this study lies.

## Author contributions

JZ: Writing – original draft. GL: Writing – review & editing, Data curation. XY: Writing – review & editing. CZ: Writing – review & editing, Data curation. BH: Writing – review & editing. MJ: Writing – review & editing, Methodology.
